# Molecular detection and genotyping of enteroviruses from CSF samples of patients with suspected sepsis-like illness and/or aseptic meningitis from 2012 to 2015 in West Bank, Palestine

**DOI:** 10.1371/journal.pone.0172357

**Published:** 2017-02-22

**Authors:** Kamal Dumaidi, Amer Al-Jawabreh

**Affiliations:** 1 Department of Medical Laboratory Sciences, Faculty of Allied Health Sciences, Arab American University in Jenin, Jenin, Palestine; 2 Al-Quds Public Health Society, Jerusalem, Palestine; Aga Khan University Hospital Nairobi, KENYA

## Abstract

**Background:**

Human enteroviruses (HEVs) are the most frequently reported cause of aseptic meningitis with or without CSF pleocytosis in childhood. Rapid detection and genotype of HEVs is essential to determine the causative agent and variant causing sepsis-like illness and/or aseptic meningitis.

**Aim:**

To investigate the molecular epidemiology of enteroviruses (EVs) among patients with sepsis-like illness and/or aseptic meningitis admitted to three major hospitals in West Bank, Palestine from 2012 to 2015.

**Methods:**

During the study period, 356 CSF samples were collected from patients with sepsis-like illness and/or aseptic meningitis. Two RT-nested PCR assays targeting a partial part of 5'UTR for direct diagnosis and the VP1 region for genotyping by sequence analysis of the viral genome were used.

**Results:**

HEV RNA was detected in 66 of 356 (18.5%) of CSF samples. Age distribution showed that 64% (42/66) were infants (<1 year), 18% were children between 1 and 5 years old, 12% were children between 5 and 10 years old, and 6% were more than 10 years old. Of the 66 EV cases, 12 were successfully genotyped. Five different EV genotypes were identified. All of them belonged to HEV-B species. The study showed that echovirus 6 genotype accounted for 42% of the sequenced cases. The HEV infections in the present study tended to show slight seasonal pattern with more cases occurring during spring and summer, yet still significant numbers were also reported in fall and winter seasons.

**Conclusion:**

HEV was isolated from a significant number of children with sepsis-like illness and/or aseptic meningitis. In addition, the molecular method utilized for direct diagnosis and genotyping of HEV from CSF revealed that more than one HEV type circulated in the West Bank, Palestine during the study period.

## Introduction

Human enteroviruses (HEVs) are non-enveloped viruses with positive sense single-stranded RNA genomes. Enterovirus genus is a member of the *Picornaviridae* family with more than 100 distinct genotypes across four species (human enterovirus A to D) based on a partial or whole sequence analysis of the VP1 region of the viral genome [1, 2]. Human enterovirus infections are usually transmitted via the fecal-oral route. Although, many infections are asymptomatic, they still cause a diverse range of clinical manifestations including acute flaccid paralysis, myocarditis, neonatal sepsis, febrile illness, haemorrhagic conjunctivitis, hand-foot-and-mouth disease (HFMD), encephalitis and aseptic meningitis [2, 3]. During the last 2 to 3 decades and in many parts of the world, HEVs have been considered the major causative agents of aseptic meningitis, a meningeal inflammation not caused by an identifiable bacterial pathogen in the cerebrospinal fluid (CSF) and with CSF pleocytosis, CSF white blood cells (WBC) count (/mm^3^) of >35 for age <30 days, >25 for age 30–60 days, and >5 for age 60 days[4]. Furthermore, a significant number of enteroviral meningitis without CSF pleocytosis have been detected from CSF samples, especially in young infant using Reverse transcription-PCR (RT-PCR). [5, 6]. Numerous HEV types have been detected and identified from sporadic and outbreak forms of the diseases [2, 7].

Human enterovirus B species including coxsackievirus "CV" A9 and CVB1-CVB6; echovirus "E" 1–7, 9, 11–27, and 29–33; enteroviruses "EV" 69, 73–75, 77–88, 97, 100, and 101 have been isolated and identified from patients with aseptic meningitis. Recently, E30 was reported as one of the most frequently detected HEVs in European, American, Asian and African countries either in epidemic or endemic form of aseptic meningitis and sepsis- like illness. Other less frequent but significant HEVs types had also been reported including E4, E9, E6, enterovirus 71, CVA9, CVB3 and CVB5 [2, 8–12].

Currently, polymerase chain reaction (PCR) assays targeting the 5' un-translated region (5'-UTR) and sequence analysis of a part or the whole VP1 as well as VP4-VP2 regions have replaced the classical methods such as cell culture and sero-neutralization for the diagnosis and genotyping of HEVs. This method has been proven to be more rapid and more sensitive than virus isolation for detecting and typing of HEVs directly from CSF samples [13, 14]

HEV sepsis-like illness and/or aseptic meningitis usually has a benign course and treatment options are limited. However, HEVs surveillance is crucial for early identification of such cases to avoid further testing and inappropriate treatment.

In Palestine, the incidence rate of sepsis-like illness and/or viral meningitis in 2014 was 6.8 per 100,000 in the West Bank and 247 per 100,000 in the Gaza Strip [15]. However, in Palestine as well as in most of the neighboring Arab countries there is little information on the prevalence and the serotypes of HEV causing sepsis-like illness and/or aseptic meningitis. Therefore, this study aimed to investigate HEV epidemiology among patients with sepsis-like illness and/or aseptic meningitis with or without CSF pleocytosis admitted to three hospitals in the West Bank, Palestine from 2012 to 2015.

## Materials and methods

### Patients and clinical samples

From July 1, 2012 to November 31, 2015, 356 CSF samples were collected from patients admitted to hospitals in the West Bank, Palestine. All patients were highly suspected of having sepsis like illness and/or aseptic meningitis. The patients were clinically diagnosed by physicians in the aforementioned hospitals and CSF samples were collected by lumbar puncture. All CSF samples were examined by culture and gram stain for common bacterial pathogens in hospital microbiology departments and proved to be negative. Samples were transferred to the Virology Laboratory at the Medical Laboratory Sciences Department, Faculty of Allied Medical Sciences, Arab American University Jenin and stored at -20°C until tested.

The patients’ demographic data including age, sex and place of residence, as well as the date of onset of symptoms, clinical history, and CSF laboratory test results were retrieved from the patients’ files. The study was approved by the Ministry of Health in Palestine under reference number ATM/125/2013.

### Viral RNA extraction

The viral RNA was extracted from 200μl of the CSF samples, using a QIAamp MiniElute Virus spin kit for the viral RNA extraction (QIAGEN,) according to the manufacturer’s instructions. The final volume of the RNA elute was 35μl.

### HEV diagnosis, genotyping and phylogenetic analysis

Screening for enteroviral RNA was performed using two sets of primers targeting the 5' UTR as described previously [16] with few modifications. Synthesis of the cDNA and the first round amplification of the RT-PCR was carried out in 25μl- reaction mixture containing 4 μl viral RNA extraction, 10 U Reverse transcriptase (AMV), 10 pmol of the outer primers (P1 and P2) and 12.5 μl of PCR Reddy master mix (Thermo Scientific). Two microliters from the first round were further amplified in 25 μl reaction mixture containing 10 pmol of the inner primers (P2 and P3) and 12.5 μl of the PCR master mix (Thermo Scientific). In each PCR run, negative and positive controls were included. Five microliters of PCR product were analyzed by electrophoresis on a 2% agarose gel containing ethidium bromide and were visualized using the Gel Doc System 2000 (Bio-Rad Laboratories-Segrate, Milan, Italy). A band of 203 bp indicated a positive result.

The portion of VP1 (AminoTerminal Part of VP1) was amplified using two degenerated primer sets as described previously [17]. Briefly, the synthesis of the cDNA and the first round amplification of the RT-PCR was performed in 25 μl reaction mixture containing 10 pmol of two outer primers (EV-F and EV-R), 10 U AMV and 12.5 μl PCR master mix (Thermo Scientific). Two microliters from the first round was further amplified in 25μl reaction mixture containing 10 pmol of the inner two primers (ENTNES-F and ENTNES-R) and 12.5 μl PCR master mix (Thermo Scientific). In each PCR run, negative and positive controls were used. A band of 400 bp visualized on agarose gel electrophoresis indicated a positive result. The PCR products were purified and sequenced in forward and reverse directions. HEV identity search was conducted using GenBank Basic Local Alignment Search Tool (BLAST) http://www.ncbi.nlm.nih.gov/BLAST/Blast.cgi. The DNA sequencing data reported in this study have been submitted to GenBank under the accession numbers.

The 12 EV study sequences were aligned with the corresponding EV prototype and other EV strains that showed high identity (95%) over the entire length of the sequenced amplicons that had be retrieved from the GeneBank. A phylogenetic tree was constructed by maximum likelihood with 1000 replicates using MEGA 7 [18].

### Statistical analysis

Data analysis for frequency tables, association, and graphs was conducted using the Epi Info statistical package (CDC free-software). MEGA 7 was used for constructing the phylogenetic tree.

## Results

### Clinical features and demographic data

A total of 356 CSF samples were collected from hospitalized patients in 3 major hospitals in the West Bank area of Palestine: Jenin Government Hospital in the city of Jenin in the north, Rafidia Government Hospital in Nablus in the north and Al-Khalil Government Hospital in Al-Khalil in the south. The CSF samples were collected over four years from 2012 to 2015. Of the patients, 207 (58%) were males and 149 (42%) were females. Approximately 62% of the patients were infants less than one year, 20% were children between 1 and 5 years old and the rest were over 5 years old. Approximately 92% of the patients (327/356) suffered from fever at the time of hospital admission (Table 1).

**Table 1:** Demographic data and clinical manifestations of the study population.

Age group	No. (%)	Male: Female	Fever	Headache	Neck Stiffness	Photophobia	Diarrhea
<1	221(62.1)	130:91	202	21	5	5	28
1–5	73 (20.5)	42:31	69	9	2	3	10
5–10	30 (8.4)	19:11	26	14	3	0	1
>10	32 (9)	16:16	30	19	4	4	4
**Total**	**356**	**207:149**	**327**	**63**	**14**	**12**	**43**

### Molecular identification and genotyping of EV

**Fig 1:**
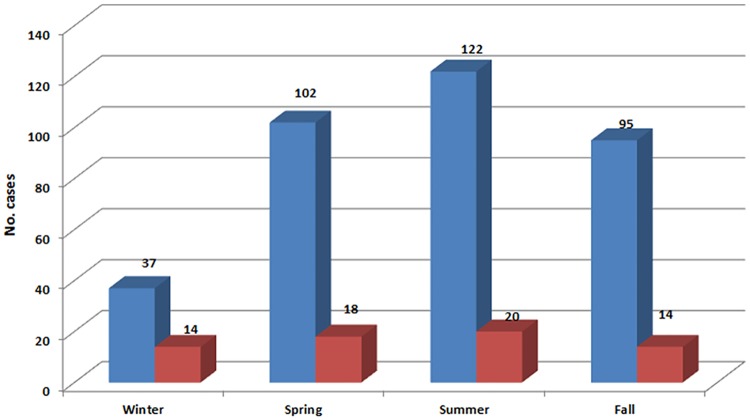
Distribution of HEV cases (red bars) by season compared to total number of cases (blue bars).

**Fig 2:**
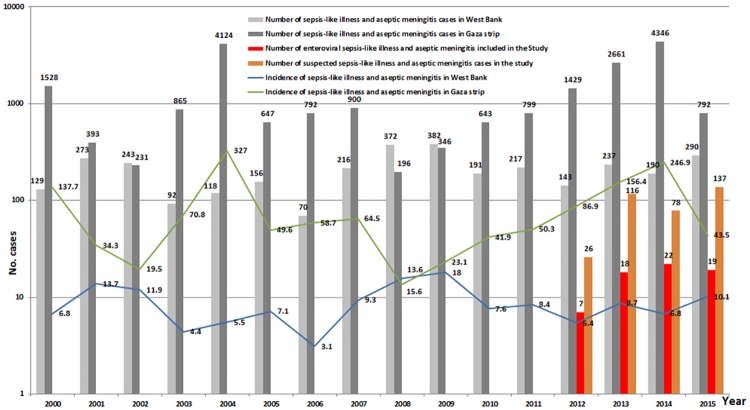
HEV cases included in the study (2012–2015) with Ministry of Health-reported sepsis like illness and/or viral meningitis cases and incidence rates (2000–2015).

Using the nested RT-PCR targeting 5'UTR, 66 out of 356 (18.5%) were positive for enteroviral-RNA. Around two-thirds (42/66) were male. The age distribution was 64% (42/66) infants (<1 yr), 18% children between 1 and 5 years of age, 12% children between 5 and 10 years of age and 6% >10 years old. EV infection in Palestine tended to show slight seasonal pattern with 58% (38/66) of the cases reported during spring and summer (Fig 1). The infection rate is highest in the southern city of Al-Khalil (42%) followed by the northern cities of Nablus and Jenin; 21% and 15%, respectively, with 2014 as the peak year (Fig 2). Of the 66 HEV cases, 12 were successfully genotyped and sequenced. Seven different EV genotypes were identified (Table 2). All genotypes belonged to HEV-B species. The study showed that the echovirus 6 genotype accounted for 42% of the sequenced cases.

**Table 2:** Distribution of HEV genotypes.

Laboratory Code	Accession Number	Genotype	Year	Age Group (in years)
670 C (control)	KX059434	Echovirus 13	2007	<1
J50	KX059435	Coxsackievirus B5	2013	<1
N165	KX059436	Echovirus 14	2015	<1
N175	KX059437	Echovirus 6	2015	1–5
N180	KX059438	Echovirus 9	2015	<1
N184	KX059439	Echovirus 6	2015	>10
N185	KX059440	Echovirus 6	2015	<1
J31	X059441	Echovirus 6	2015	<1
J39	KX059442	Echovirus 6	2015	<1
J104	KX059443	Echovirus 30	2013	5–10
J103	KX059444	Echovirus 16	2015	>10
J59	KX059445	Coxsackievirus B5	2013	<1

HEV was found to be statistically associated with pleocytosis (P<0.05) with 38% in HEV cases compared to 17% in non-HEV cases. However, still 62% (31/50) of the HEV cases did not have pleocytosis. Of the study population, approximately 21% (64/307) showed pleocytosis with a mean of 35.4 WBC/mm^3^ and a range of 0–1300 WBC/mm^3^.

### Phylogeny of HEV strains based on VP1

**Fig 3:**
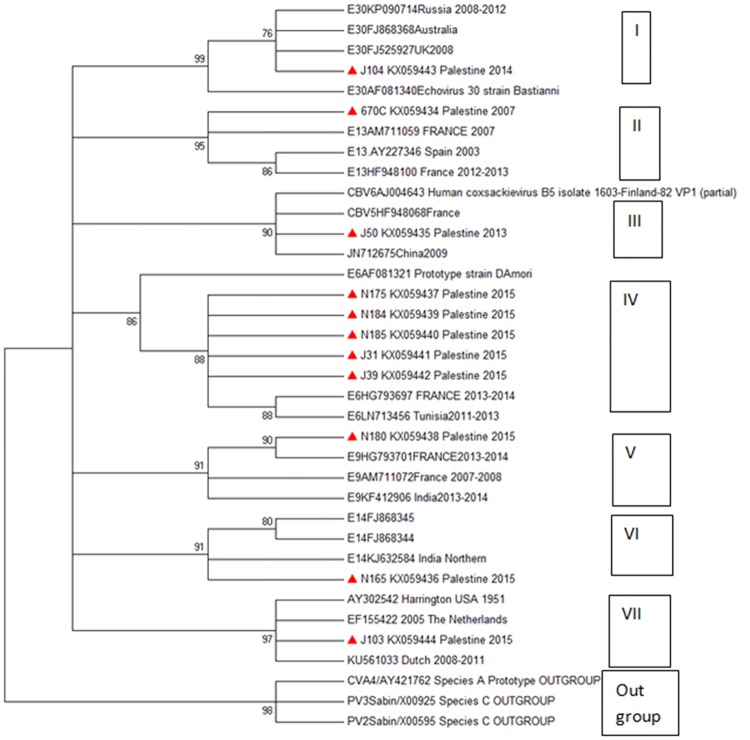
Phylogenetic consensus tree. The tree depicts the viral capsid protein 1 (VP1) gene sequences for the 12 HEV isolates in Palestine (red triangle and tagged by their laboratory code and Accession number) along with 22 isolates from GenBank belonging to different geographical areas and genotypes. The tree was reconstructed by the maximum likelihood statistical method and based on the Tamura-Nei method. A bootstrap validation of 1000 replicates and a cut-off value for consensus tree of 75% were used. Polio virus-Sabin strains and HEV 68 were used as an out group.

The phylogenetic analysis revealed 7 main HEV genetic clusters with a consensus of 86 to 99%. Five of the 12 Palestinian strains clustered in cluster IV which contained HEV genotypes from France and Tunisia. The study strains in cluster IV were isolated in the same year, 2015. The 3 strains isolated in 2015 showed dispersal clustering. The other six strains were distributed equally to the other 6 clusters. The clustering did not follow a geographical pattern (Fig 3).

## Discussion

HEVs are widely recognized as the major etiological agent of aseptic meningitis, which is caused by HEVs in 48–95% of cases in which a causative virus is identified [19, 20]. Rapid HEV identification and genotyping is essential to determine the variants that may cause large meningitis outbreaks and/or those associated with unusual clinical presentations.

Most HEV sepsis like illness and aseptic meningitis patients experience headache, fever, vomiting, and neck stiffness. Some patients may also exhibit symptoms of cold, altered mental status and seizures [2, 11]. Fever, headache, diarrhea, vomiting and neck stiffness were the most reported symptoms identified among our patients, which is consistent with the findings of other studies. In the present study, fever was the most common symptom in most patients (327/356; 92%), which is in congruence with previous studies [11, 21–23]. This is due to the fact that most of the patients are children under 1 year old who do not complain and whose temperature is measured regularly.

Infants with HEV sepsis-like illness and/or aseptic meningitis without CSF pleocytosis were identified in this study (60%). This high percentage of cases of HEV without pleocytosis is in congruence with other studies [24, 25], but in contradiction with others [5, 6]. This discrepancy could be due to different CSF pleocytosis definitions adopted and the age of the study samples.

The prevalence of HEV sepsis like illness and/or meningitis (66/356–18.5%) by RT-nPCR targeting the 5'-UTR in the present study is consistent with several previous studies from Kuwait in the Middle East (24%), China (19.2%) and Greece (15.4%) [7, 11, 26]. However, lower prevalence was reported from other Middle Eastern countries such as Jordan (8.2%), Tunisia (5%) and Spain (4.6%) [27–29]. On the contrary, a higher prevalence has been reported in several aseptic meningitis outbreaks of different HEV genotypes [8, 10]. The discrepancy in prevalence between the different studies may be due to the sensitivity of the method used for virus isolation or identification, type of sample used, the study population (infant, children and adult), gender and whether the data were from sporadic cases of sepsis like illness and/or aseptic meningitis, regular surveillance or an outbreak.

Our results revealed that 58% of the HEV cases were detected in spring and summer, yet 42% of cases were still detected in fall and winter. These results were consistent with the slight seasonality of HEV infections in temperate climates. Such a result highlights the importance of considering HEV infection that occurs throughout the year and not only in spring and summer [7, 30].

The majority of HEV cases in this study were children less than 1 year old (64%). This finding is consistent with several previous studies which showed that the majority of HEV sepsis like illness and/or aseptic meningitis meningitis cases occurred in children and mainly in those under 1 year old [9, 11, 23]. The reason behind this finding may be due to the immature immune system in children and infants, which make them more susceptible to enteroviral infections. Although enteroviral infections in teens and adults are less common, they should not be overlooked [9, 11, 23].

The method of HEV typing used in this study succeeded in 12 cases out of 66 (18.2%). The low rate of direct HEV typing from the CSF was in agreement with the study conducted by Krasota *et al*., that showed a success rate of direct HEV typing ranging from 19.5% to 54.1% using their own and other previously developed assays [30–34]. On the other hand, Thoelen *et al*., (2003) in Belgium and Molet *et al*., (2016) in France succeed in typing 65% and 87.8% of the HEV from CSF and other clinical biological samples respectively. This low rate of success in typing can be attributed to the high variability of the VP1 region of the HEV genome and the low sensitivity and specificity of the degenerated primers used. Furthermore, the low genotyping rate in our study could be attributed to the use of only two primer sets out of several sets described previously by Thoelen *et al*., [17], presence of PCR inhibitors and/or degradation of the viral RNA due to repetitive thaw- freeze cycles.

The enteroviruses that were typed in this study by sequencing the partial VP1 region of the EV genome were found to belong to HEV species B (E6, E9, E30, E13, E16, E14 and CVB5). The HEV-B species are shown to be associated most commonly with aseptic meningitis [32, 35]. In addition, HEV genotypes—especially E6, E9, E13 and E30 and CVB5—have been reported in both sporadic and outbreak aseptic meningitis cases in Kuwait, Greece, Australia, Korea, South Africa and Russia [9–11, 23, 26, 31]. The low detection rate of E13, E30 and E9 could be due to the absence of outbreaks, because these types were not prevalent in the West Bank, Palestine during the study period or because of the low success rate of HEV typing (18%). This study also found rare HEV types including E16 and E14. A pattern of distribution similar to that in our study has been reported in India, Russia and Korea [10, 18, 31].

In conclusion, this study showed that HEV is prevalent among children with sepsis-like illness and/or aseptic meningitis. Accurate diagnosis of HEV sepsis-like illness and/or aseptic meningitis will reduce antibiotic and antiviral use as well as save precious life-saving time lost due to inaccurate diagnosis. Furthermore, this study revealed that there is more than one HEV type circulating in the West Bank, Palestine. Finally, implementing molecular methods for diagnosis and typing of HEV will provide valuable epidemiological tools that can be used to identify and monitor HEV from sepsis- like illness and from sporadic and outbreaks of aseptic meningitis.
